# A System for In-Line 3D Inspection without Hidden Surfaces

**DOI:** 10.3390/s18092993

**Published:** 2018-09-07

**Authors:** Juan-Carlos Perez-Cortes, Alberto J. Perez, Sergio Saez-Barona, Jose-Luis Guardiola, Ismael Salvador

**Affiliations:** 1 Instituto Tecnológico de Informática( ITI), Universidad Politècnica de València, 46022 Valencia, Spain; ssaez@iti.upv.es (S.S.-B.); joguagar@iti.upv.es (J.-L.G.); issalig@iti.es (I.S.); 2 Departamento de Informática de Sistemas y Computadores (DISCA), Universidad Politècnica de València, 46022 Valencia, Spain

**Keywords:** octree carving, shape from silhouette, visual hull, 3D metrics, 3D reconstruction, camera calibration

## Abstract

This work presents a 3D scanner able to reconstruct a complete object without occlusions, including its surface appearance. The technique presents a number of differences in relation to current scanners: it does not require mechanical handling like robot arms or spinning plates, it is free of occlusions since the scanned part is not resting on any surface and, unlike stereo-based methods, the object does not need to have visual singularities on its surface. This system, among other applications, allows its integration in production lines that require the inspection of a large volume of parts or products, especially if there is an important variability of the objects to be inspected, since there is no mechanical manipulation. The scanner consists of a variable number of industrial quality cameras conveniently distributed so that they can capture all the surfaces of the object without any blind spot. The object is dropped through the common visual field of all the cameras, so no surface or tool occludes the views that are captured simultaneously when the part is in the center of the visible volume. A carving procedure that uses the silhouettes segmented from each image gives rise to a volumetric representation and, by means of isosurface generation techniques, to a 3D model. These techniques have certain limitations on the reconstruction of object regions with particular geometric configurations. Estimating the inherent maximum error in each area is important to bound the precision of the reconstruction. A number of experiments are presented reporting the differences between ideal and reconstructed objects in the system.

## 1. Introduction

Fast and complete methods for 3D reconstruction of industrial parts or other objects in a production line are needed when verification of global geometric attributes or full surface quality checks are required for a 100% inspection of high volume productions.

Industrial inspection is an important field which requires fast and accurate systems. Advanced 3D metrology equipment is often designed to be located in the metrology lab and not on the production floor. The final inspection throughput is thus bounded by the sum of the times required to sample the production, the movement of the parts to the lab and the the measurement itself. This increases production costs and involves skilled human resources who can operate laboratory equipment and interpret the measurement results. The complexity of the CAD designs rises as the manufacturing technologies improve, and the production costs, at the same time, are decreasing due to global competition.

In products that are composed of many parts that must be assembled, often many measurements must be performed to cover all the attachment points and small deviations can affect the process with a result of lost productivity or, even worse, of defects in the final product.

Traditional equipment such as coordinate measuring machines (CMM) are accurate but slow, causing bottlenecks and reducing the percentage of the products that are sampled. To obtain 100% of the production inspected, the system must be designed to follow typical manufacturing rates and be inserted in the production line. Non-contact measurement systems like 3D scanners have the potential to become a useful part of the inspection process.

However, nowadays, the typical inspection systems in industry are still 2D and so-called $2\frac{1,2}{D}$ systems, which are based only on overhead, frontal or lateral views, leaving hidden regions of the object that cannot be analyzed, and more thorough 3D systems that are able to capture a larger proportion of the object surface require long processes with sequential image acquisition on turntable systems using stereoscopy, laser beams or structured light [1,2,3]. Robots that move the object in front of a sensor or move the scanner to follow the shape of the object are also available [4,5]. Other industrial solutions such as Avizo Inspect [6] use more sophisticated equipment and time-consuming procedures to apply computed tomography to obtain a full reconstruction of an object.

**Figure 1:**
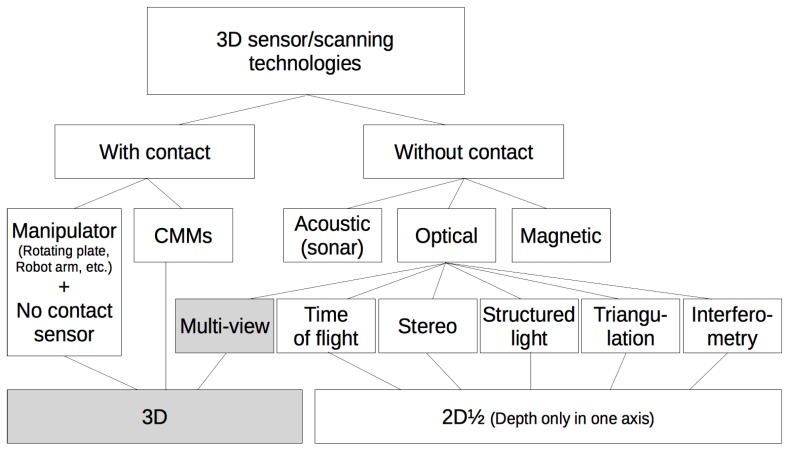
Taxonomy of current 3D scanning methods. The technology proposed in this paper belongs to the highlighted categories.

In [7], a survey of recent non-contact 3D scanners with a classification of their technologies and an overview from a manufacturing perspective is presented along with potential manufacturing applications and the practical requirements of typical industrial tasks. In [8], the resulting data files obtained from various types of 3D scanners are analyzed. In Figure 1, a taxonomy of the existing 3D scanning technologies is shown.

In order to get all the views at the same time, we propose a single-shot multi-camera acquisition system. The system for this task is intended to reach real-time operation with latencies of a few seconds and productivities of several objects per second using straightforward parallel processing. Preliminary descriptions of the ideas and early prototypes of this system were presented in [9,10]. Two patents have also been registered [11,12].

**Figure 2:**
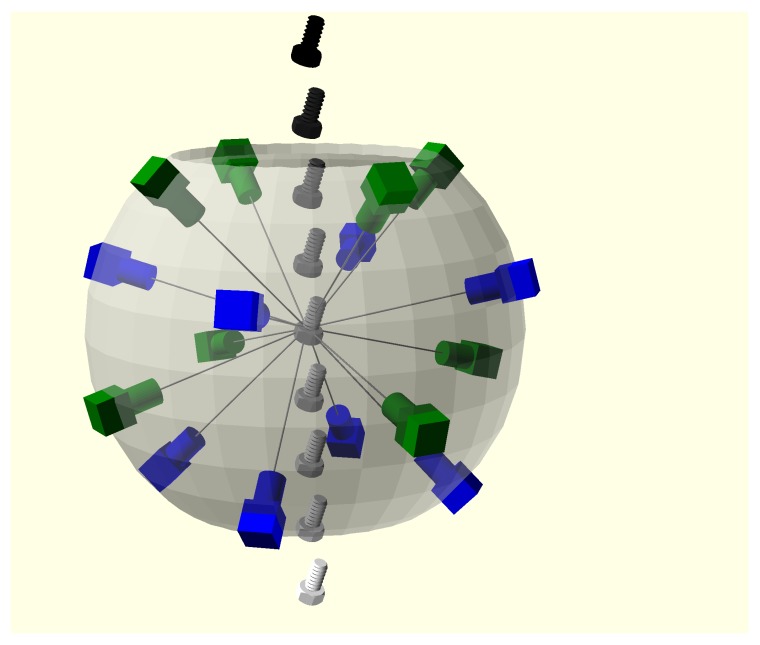
Camera configuration. Objects fall free through the device, being captured in the center where the camera views converge.

The prototypes employ a multi-view scheme, with 16 synchronized cameras conveniently distributed on the faces of a polyhedron (see Figure 2). The object to scan falls through the vertical axis of the structure and is captured exactly when it is in its center, being in the field of all the cameras at the same time. Since the object is in free fall, there is no handling tool or surface which hides any part of it. Thus, the whole object can be acquired with just one shot and no robot arm or handler has to be designed, adapted and programmed to manipulate and present the part to the cameras or sensing devices. This makes the system especially useful for quality inspection in production lines that are manufacturing different items, even in an variable or unknown sequence. The software can recognize the reconstructed part and apply the relevant quality checks without any tool change or mechanical reconfiguration.

Other mechanical options besides a simple fall can be devised to achieve the acquisition of the object in the system with no support surface. We are now testing linear motors that impulse the object to the center of the device and gently recover it with no impact.

An adequate diffuse illumination is important to avoid shadows and reflections. It is obtained from high-power LEDs also attached to the camera supports or to the inner faces of the polyhedron and conveniently scattered by reflection or by a polycarbonate sphere that acts as a diffuser and a background image for all the cameras.

**Figure 3:**
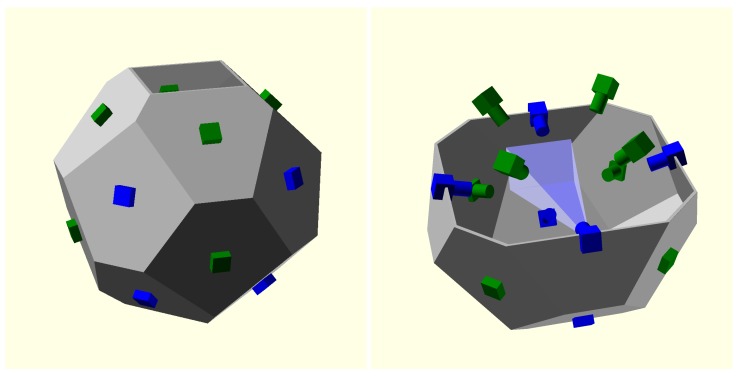
(**Left**) polyhedron supporting the cameras, this is a more efficient industrial design than a sphere; (**Right**) the camera distribution guarantees that each camera field of view does not contain other cameras.

The polyhedron used has 16 faces, none of which are parallel to any other to guarantee that no camera is seen in the field of view of any other camera (see Figure 3), to obtain smooth backgrounds and the maximum intersection volume.

**Figure A1:**
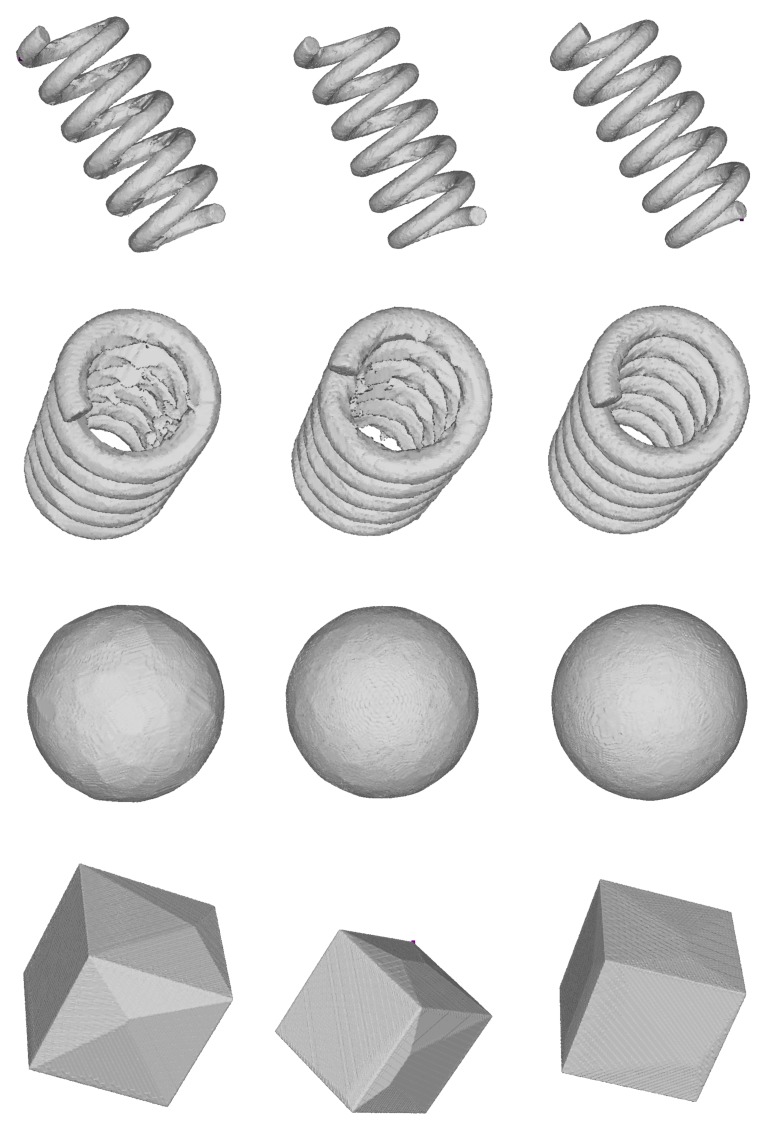
Examples of object reconstruction improvement with increasing numbers of cameras; (from **Left** to **Right**) 16 cameras, 24 cameras, and 48 cameras.

After a number of simulation analyses on different industrial objects to characterize the inspection quality and tolerances that can be expected from the system in various tasks, we found that the larger the number of cameras used, the better the results were (see Section 5 and Figure A1 in Appendix A). We decided to use 16 views due to the increasingly high costs of the hardware (cameras, lenses, mechanics and interface electronics, along with the computer power needed) and of the reconstruction and inspection algorithms. We plan to build systems with less/more cameras if we face less/more demanding practical applications.

**Figure 4:**
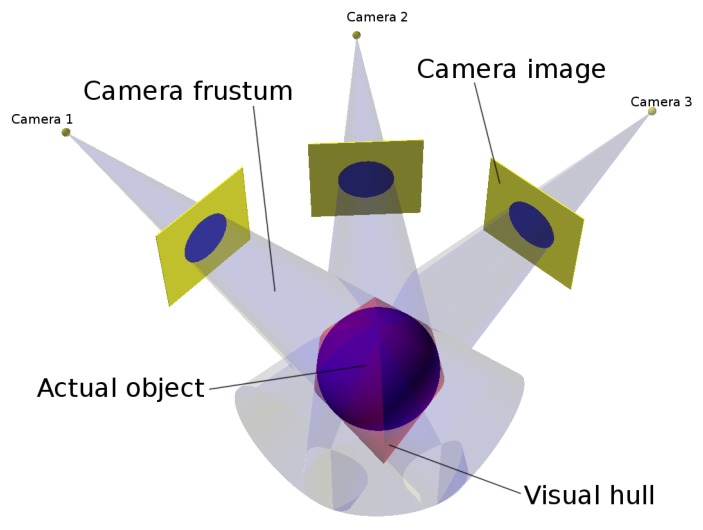
Visual hull.

A shape-from-silhouettes approach [13] has been used to obtain a 3D model. Thus, the 3D object shape is built by the intersection of the pixel frustums formed by back-projecting the pixels marked as “object” according to the silhouettes in the corresponding images. (A frustum is a truncation with parallel planes of the pyramid of vision: one of the planes is the surface of a pixel on the camera sensor and the other is whether the area sensed on the object that is being imaged or the corresponding area on the background). The reconstructed 3D object shape is not guaranteed to be the same as the original object since concave surface regions can never be distinguished using silhouette information alone (see Figure 4).

The visual hull [14] is the maximal shape consistent with silhouettes of an object as seen from any viewpoint in a given region that can be constructed by intersecting the frustums generated by back-projecting the object silhouettes of a given set of views. The proposed visual hull computation method belongs to a volumetric method based on the polygonization of an octree structure [15] by using a marching cubes algorithm. (An octree is a tree structure with internal nodes having eight children each that are used to represent a 3D object by partitioning the space by recursively subdividing a volume into eight octants).

For objects with sufficient texture, stereo (photoconsistency based) methods can be applied to improve the 3D model beyond the visual hull. Good results have been obtained in other applications using patch-based multiview stereo algorithms [16,17]. Other options to obtain a refined model is to add a scheme of structured light [18] or fringe projection for Fourier or phase-shifting profilometry [19].

We do not address in this paper the defect detection process which has to be applied after reconstruction to compare the test object against a reference model. Recent works in this area, like [20] or [21], show promising results even in difficult tasks.

This paper is organized as follows. Section 2 describes the 3D model generation process. Section 3 explains the camera calibration approach developed for this system. Section 4 presents a more detailed description of the implemented hardware. Section 5 reports the experiments performed and shows examples of reconstructed objects. Finally, conclusions and future work are presented in Section 7 and Section 8.

## 2. Multi-View 3D Model Generation

On this section, we study the process of 3D model generation from multiple views. Since these views are obtained from simultaneous captures of a falling object, a unique diffuse illumination setting must be used for all the cameras.

### 2.1. Image Segmentation

**Figure 5:**
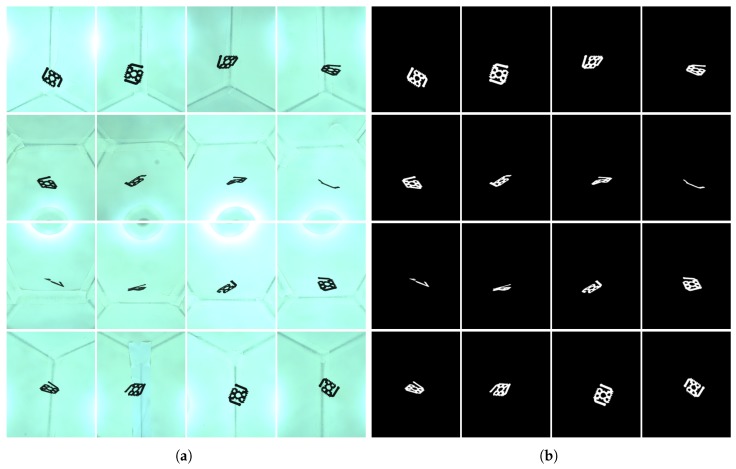
First steps in the 3D model generation: (**a**) image capture; and (**b**) silhouette segmentation.

The first step is to segment camera images to obtain the object silhouette for each view. Figure 5a shows a capture example.

Image segmentation is an important step where we need to decide on the membership of each pixel, whether it belongs to the object class or to the background class.

Two types of illumination schemes have been tested: reflective backgrounds illuminated with light sources located around each camera, or a more uniform illumination using evenly distributed light sources in the background and a diffusion system (i.e., a translucent sphere).

In both cases, a reference capture with no object in the image is carried out at the beginning of each session to obtain a set of background images that are used to make the segmentation process more robust and independent on changes on the illumination characteristics or on uneven backgrounds with non-uniform illumination, internal structures, etc. (see Figure 5a).

When a new set of images is acquired, we check every pixel of the captured images and compare it to the corresponding background pixel. Due to noise, shadows and other reasons, background pixels usually have difference values higher than 0 and therefore a threshold has to be used to decide if a pixel belongs to the object or to the background (see Figure 5b).

**Figure 6:**
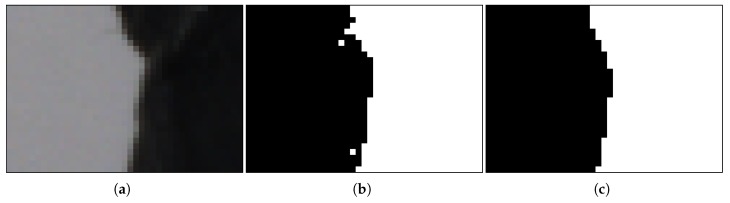
Steps of the image segmentation process: (**a**) original image; (**b**) segmented; and (**c**) smoothed.

To deal with the remaining artifacts appearing in the silhouette borders (Figure 6b), the segmented images were smoothed using a 9 × 9 Gaussian filter before binarization [22] (Figure 6c). The segmentation threshold and the Gaussian filter parameters were adjusted to minimize the size error between reconstructions and reference objects.

### 2.2. 3D Reconstruction through Octree Carving

**Figure 7:**
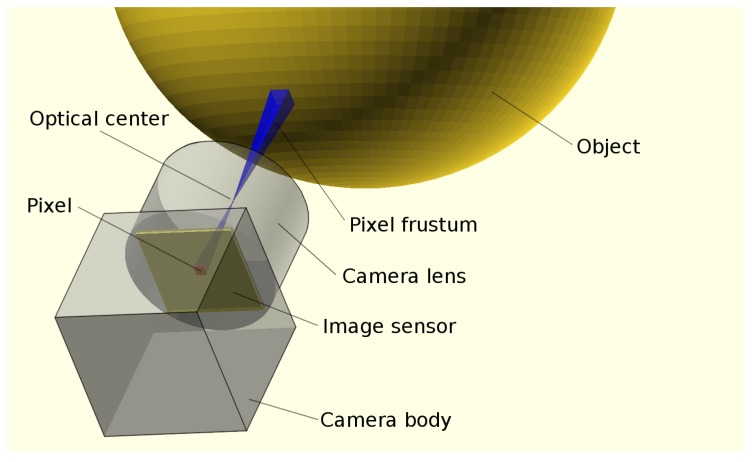
Pixel frustum.

**Figure 8:**
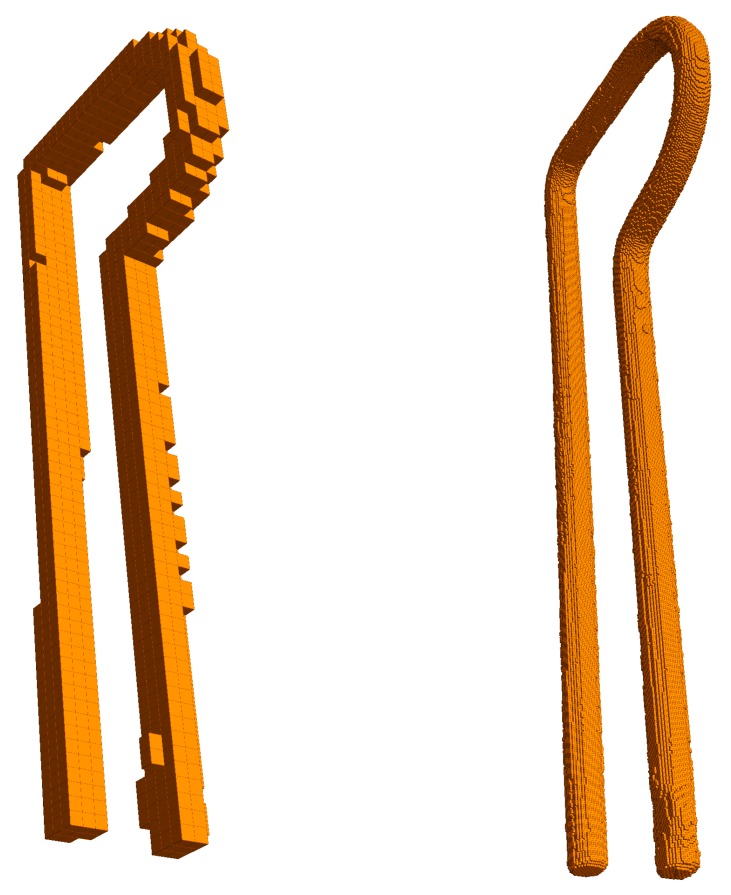
Octree generated for two different depths. (**Left**): 7 levels. (**Right**): 10 levels.

After the segmentation phase, a square-based cone or pyramid is projected from each pixel of each camera, giving rise to a Right frustum (a frustum that truncates a Right pyramid, that is a pyramid with its apex directly above the centroid of its base) with the pixel surface on the sensor as its base and the distance to the background as its height (see Figure 7). This frustum defines a 3D volume, and the intersection of the corresponding volumes of the pixels marked as “object” after segmentation from all the cameras is computed to build an octree as a result, according to [15,23]. The process can be seen as carving an initial 3D cube containing the common area viewed by the cameras by mean of those frustums. The space of the initial cube is divided into eight sub-cubes (octants). A sub-cube can be removed if it is outside at least one frustum. The process starts again with the subdivision of each remaining sub-cube until a specified depth (see Figure 8). The result of this process is an octree structure containing the remaining sub-cubes.

In other words, starting from a large first voxel that comprises the whole acquisition space, the carving algorithm checks each octant of the voxel to determine if it lies completely inside all silhouettes. In that case, it is part of the resulting octree. If it lies outside any silhouette, it is removed. If it intersects one or more silhouette contours and is completely inside the others, then it is split into eight smaller voxels and recursively processed.

To optimize and improve the accuracy, data caches for the vertices and integer coordinates have been used in the algorithm implementation.

**Figure 9:**
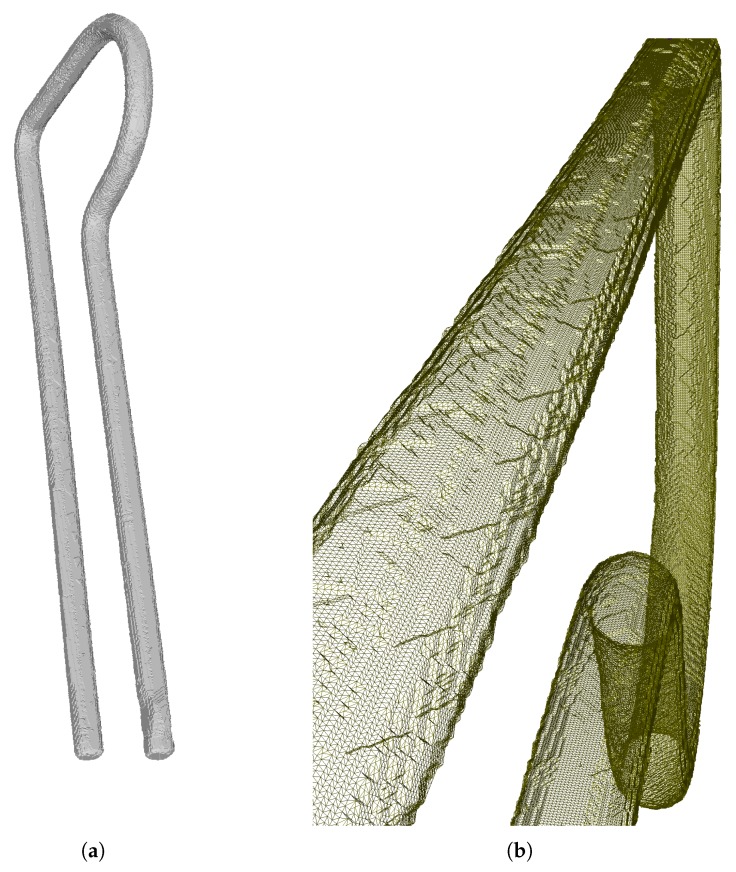
Reconstructed object.(**a**) The obtained object surface after applying the Marching Cubes algorithm. (**b**) Detail of the surface triangles.

Once the carving process has generated an octree, an isosurface algorithm is applied to obtain a 3D model (see Figure 9) using a modified version of the Marching Cubes algorithm [24] that uses fractal curves to avoid costly geometric operations and find and delete replicated points. This allows the vertices of the iso-surface to be computed only once and the resulting speedup is very significant.

The problem that must be solved by this process is the estimation of planar facets that represent the surface defined by the octree, which is a data structure that represents a volume, not a surface. The surface may cut off vertices or pass through the voxels in many complex ways. Voxel vertices may be above or below the isosurface, so the algorithm involves estimations and interpolations.

The final complete procedure gives rise to a very detailed mesh of triangles with more detail than needed according to the resolution of the silhouettes. Therefore, a simplification of that mesh is necessary. To delete small and degenerated triangles in the structure, a method adapted from [25,26] was employed. Decimation is a surface mesh simplification that involves reducing the number of faces (triangles in this case) used in a surface mesh while keeping the global shape, boundaries, and volume preserved in the highest possible degree.

### 2.3. Texture

In some applications, where visualization is involved or when the surface features are important for quality control, classification, etc., a texturing method is needed. We adapted the techniques proposed in [23], where the previous surface decimation employed allows for a better projection accuracy.

The method involves subdividing each triangle into particles which are points equi-spaced on the triangle surface. The number of particles is proportional to the triangle size. Then, to find the normal vector of each particle, a bilinear interpolation is computed with the normal vectors of the vertices of the triangle. These normal vectors are used to select the cameras with the closest optical axes, taking into account also the visibility constraints regarding hiding and surface orientation. A depth buffer accelerated via OpenGL allows a fast operation.

**Figure 10:**
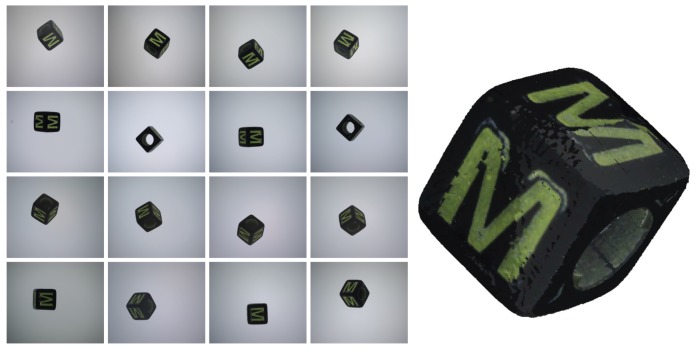
(**Left**) set of images from a capture; (**Right**) textured 3D model reconstructed using the proposed system.

Then, the pixel values of the selected cameras are combined. To weigh the contribution of each pixel, we use the dot product of the normalized camera vectors and the particle normal vectors. In Figure 10, the result of applying the texture to our example can be shown.

## 3. Multi-View Camera Calibration

All camera intrinsic parameters have been computed using a standard calibration method [27] for each camera but, in multi-camera systems, an accurate and efficient method to assure a common geometrical reference frame and optical consistency is essential. To guarantee that, a set of parameters must be computed and adjusted to define a camera model from relative correspondences between 3D points and camera pixels. The parameters obtained will then be used to define a transformation between the world and image coordinate systems.

Accurate inter-camera calibration is necessary for many common problems involving the capture of more than one image at the same time. Many works have dealt with this problem [27,28,29,30], but the particular case where several cameras need to be calibrated to a high precision while they do not have available direct lines of sight to a set of common reference points is unusual. The classical procedure works through the calibration of pairs or small sets of cameras that share visible elements in a calibration target or a scene, and proceed in a chain computation until all the cameras are calibrated. This way, a camera is locally calibrated with their neighbor cameras and indirectly with all the rest. Although that procedure works theoretically well, the result often shows accuracy and stability problems, since errors can be accumulated along the chain of computations. In the experiments with our prototypes with 16 cameras, these errors were relevant and generated conspicuously bad results.

**Figure 11:**
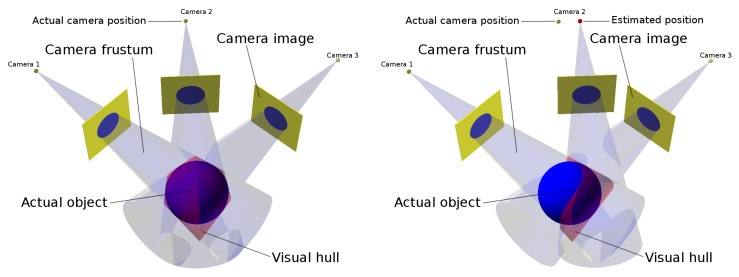
Carving result for a good calibration (**Left**) and for a wrong calibration (**Right**).

The reason is that the computation of frustum intersections during the carving step produces the loss of important parts of the object even with a minimal deviation in the estimation of the position and orientation of a camera. The volume depicted in light red in Figure 11 illustrates the different results obtained from a camera with a wrong estimated position. In Section 6.1, this can be clearly shown in Figure 19a where a bad reconstruction of a ball is obtained. An even more severe case of this problem is shown in Figure 20a where the reconstruction is very inexact and even a split of the resulting object into several unconnected segments is found.

**Figure 12:**
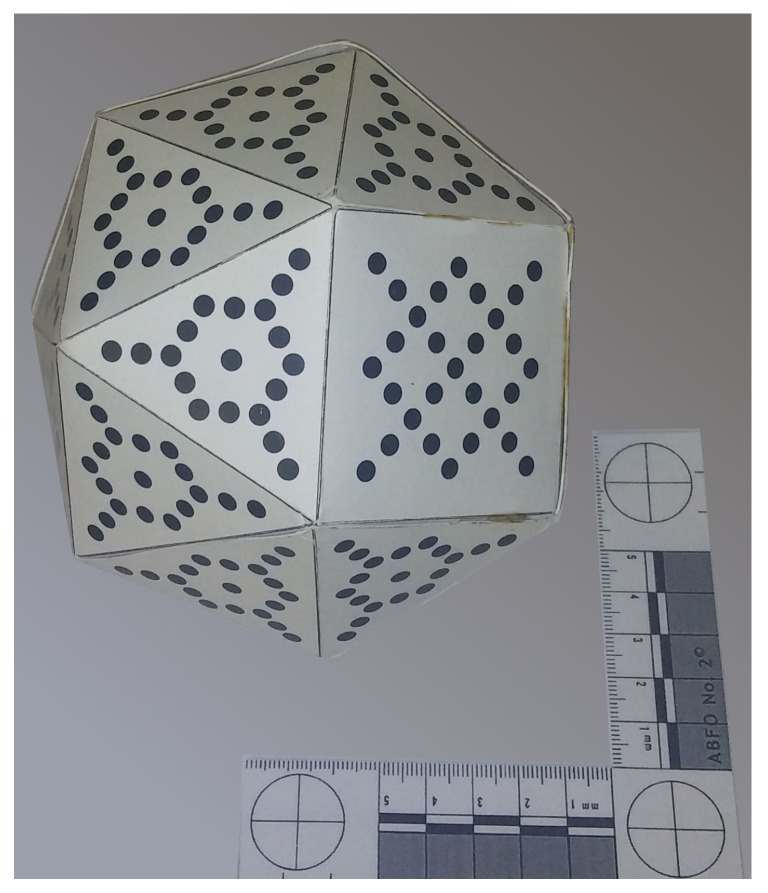
View of a 3D target used in a local camera calibration setting to compute extrinsic parameters. Each vertex of the polyhedron is intended to be facing a camera.

The problems of chained local calibrations in multiple camera configurations essentially come from the accumulation of small inconsistencies in the world coordinate systems of each camera respect to its nearest neighbors. If no global extrinsic calibration is performed, special iterative corrective procedures are needed to avoid the position and orientation errors of distant cameras that do not share any common reference point in their visual fields. We have developed and tested an alternative solution to the problem. The proposed solution avoids the error accumulation and, at the same time, the need for a complex three-dimensional target as the one shown in Figure 12.

Additionally, using a dedicated 3D target introduces difficulties associated with the precision required for its fabrication and the process of image segmentation to obtain the position of all its dots.

### 3.1. Global Extrinsic Parameter Calibration Using Spheres in Multiple Captures

**Figure 13:**
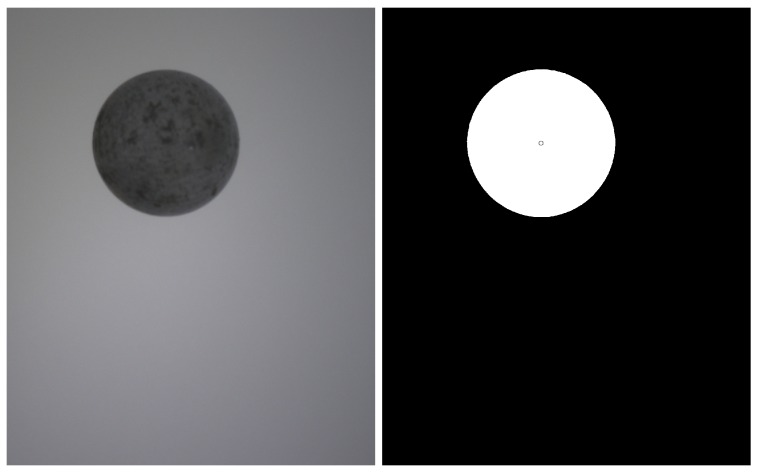
Spherical target used to calibrate. (**Left**) captured image by one of the cameras; (**Right**) segmented target showing the center.

As an alternative to the approach of local independent calibration using a 3D target, we propose to use a number of spheres captured in different (unknown) 3D positions. The centers of the circular projections on each camera of these spherical targets are used as calibration points (see Figure 13). For each target capture, 16 calibration points are obtained, all of them representing the same 3D world point.

**Figure 14:**
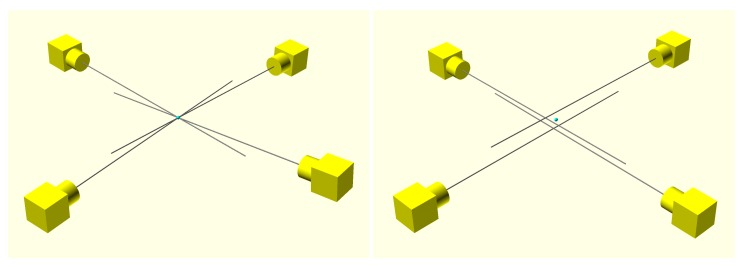
Camera projection rays of a set of calibration points in a consistent (**Left**) and in an inconsistent (**Right**) world coordinates system.

Given a set of calibration points, representing the same 3D world point, a set of camera projection rays (epipolar lines) can be computed. If the world coordinate system of the cameras is consistent, all of those rays should converge to a 3D world point. However, because of the initial inconsistencies, this will not be the case (see Figure 14).

Hence, to calibrate the extrinsic parameters of the cameras, the proposed global calibration process assumes that a 3D world point exists that minimizes the distance to all the camera projection rays.

Let $r_{i}^{\prime }=\left\{r_{i}=c_{i}+t\;d_{i}\;|\;t\in R\geq 0\right\}$ be the projection ray of camera i=1…c, where $c_{i}$ denotes the *i*-th camera optical center position and $d_{i}$ the direction vector from $c_{i}$ to the corresponding 2D projected point $p_{i}$. We will assume that all $d_{i}$ are normalized.

Let $p^{\prime }$ be the 3D point that minimizes the sum of distances to all camera projection rays. It can be computed as

(1) $$p^{\prime }=\underset{x\in R^{3}}{\mathrm{argmin}}\sum_{i=1}^{c}\delta \left(x,r_{i}\right),$$

where the minimum is computed in the set of points *x* of the 3D space and the distances are defined as

(2) $$\delta {\left(x,r_{i}\right)}^{2}=\left(x-c_{i}\right)\left[I-d_{i}{d_{i}}^{T}\right]\left(x-c_{i}\right).$$

A closed-form solution for the computation of $p^{\prime }$ is proposed in [31]

(3) $$p^{\prime }={\left[\sum_{i=1}^{c}\left[I-d_{i}{d_{i}}^{T}\right]\right]}^{-1}\sum_{i=1}^{c}\left[I-d_{i}{d_{i}}^{T}\right]c_{i}.$$

With this, Algorithm 1 is defined:
**Algorithm 1** Multi-camera extrinsic parameter calibration    **repeat**     **for** each spherical object $S_{k}$
**do**      **for** each *i*-th camera **do**     Let $p_{ki}$ be the center of $S_{k}$ projected to the camera image.     Compute and normalize $d_{i}$.      **end for**    Compute $p_{k}^{\prime }$ using Equation (3).    Set $p_{k}^{\prime }$ as the corresponding 3D coordinates of $p_{ki}$ in all cameras.     **end for**     **for** each *i*-th camera **do**    Re-calibrate extrinsic parameters.     **end for**    **until** Stop criterion attained

To re-calibrate the extrinsic parameters, a gradient descent algorithm [32] was employed using the new computed $\left\{p_{k}^{\prime },p_{ki}\right\}$ pairs for each camera.

The number of spherical objects and of iterations must be empirically set, although some geometrical requirements should be taken into account. Camera extrinsic parameters present six degrees of freedom: 3D position and 3D rotation. Therefore, a minimum of six spherical targets should be captured. Furthermore, those should be in non-coplanar space positions in order to prevent indetermination. In Section 6.1, experimental values for the number of targets and the number of iterations are discussed.

It also is important to note that an initial estimation of camera positions and camera orientations must be provided for the method to work. Since, in this application, we know the nominal geometry and measurements of our system, initialization is straightforward. In any case, a classical per-camera calibration could also be used as the initial estimation. In that case, the calibration method can be seen as a two-step procedure where the second stage is conceived as a refinement of the first one.

There are other interesting calibration algorithms that have been proposed for different tasks, like using Machine Learning as in [33].

## 4. Hardware Details

**Figure 15:**
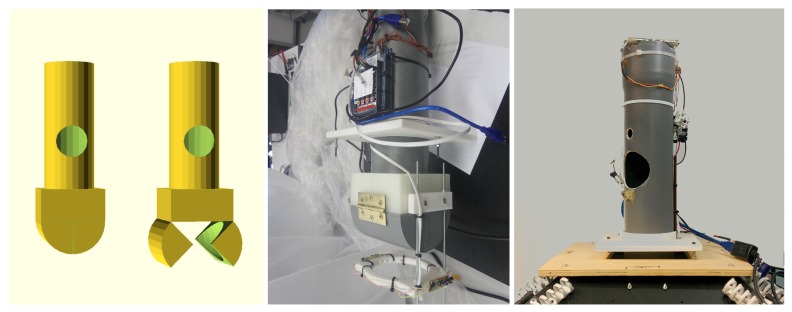
Object sequencer. (**Left**) the hatch at the bottom controls the fall of the objects; (**Middle**) the optical sensor that controls the trigger can be seen at the bottom; (**Right**) the sequencer prototype installed on top of the system, with the entry opening visible.

The image capture must be triggered synchronously by all the cameras when the object is exactly in the center of the volume defined by the intersection of the fields of view of the cameras. The objects are dropped from a conveyor into a sequencer device (see Figure 15) with a hatch and two optical detectors. One of them detects the arrival of a new object and its signal initiates the acquisition process: if the device is ready, the hatch opens releasing the object that will fall freely from a zero initial speed at that point. The second sensor is located below the hatch and detects the passing of the object, defining the initial time which, after a fixed delay, gives rise to the synchronization signal that triggers the cameras and the illumination subsystem.

**Figure 16:**
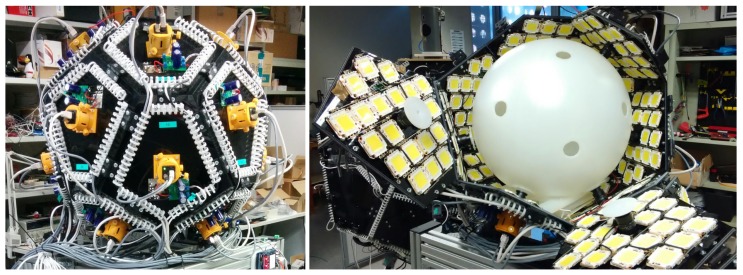
(**Left**) a prototype appearance from outside; (**Right**) the prototype disassembled to show the illumination system—in this case, with a dense distribution of LEDs and a spherical diffuser.

The illumination system is composed of high power LEDs triggered at the same time as the cameras, during a short time (comparable to the integration time of the sensors, around 40 μs in our prototypes). A high pulse of current is needed, but the average power is only of a few watts. Since the object is falling at the time of capture, the light pulse width or the integration time must be able to freeze the object image. However, a moderate to large depth of field is required to acquire the images correctly along with all the useful volume of the system; therefore, a small aperture must be used. This means that the amount of light needed is important. In Figure 16, the implementation of the illumination scenario used in the images of Figure 10 and Figure 13 can be observed.

Several prototypes have been built. The last two of them are now in operation in our laboratory. The first one has a diameter (the size of the sphere that intersects the location of the cameras) of 1000 mm and the second of 500 mm. Both have 16 cameras with global shutter sensors, effective resolution of 2448 × 2048 pixels, 2/3” sensor size, RGB (red, green and blue) square pixels of 3.45 μm side, GigE Vision interface, 23 fps and precise shutter operation (Sony XCG-CG510C industrial cameras, Sony Corporation, Tokio, Japan.). The large prototype has Fujifilm lenses (Tokio, Japan) of 25 mm focal length and the smaller one has 50 mm lenses. To obtain an adequate depth of field, the diaphragms are closed to an aperture of F16.

The size of the useful volume (maximum inspected object dimension) is 130 mm in the large prototype and 30 mm in the small one, with a measurement precision (voxel size at maximum octree depth for 3D measurements and/or projected pixel size on the surface of the object for surface measurements) of less than 0.070 mm in the large prototype and less than 0.020 mm in the smaller one.

The cameras are connected to a 10 Gb Ethernet switch with sixteen 1 Gb Ethernet links for the cameras and two 10 Gb uplinks to the computer system, which is composed of a variable number of high-performance computer nodes (depending on the variable experimental needs) running Linux. All of our software, except for the web front-ends, is written in C/C++. The computation times (latency of the inspection process) are between 1 and 10 s, depending on the complexity of the object, the number of measurements and tests performed and the precision required for the reconstruction. Many parts can be processed in parallel, giving an inspection throughput that is only bounded by the speed of the object handling mechanisms (conveyors, sequencing hardware, etc.), the camera FPS and the acquisition networking bandwidth. The specifications of the current prototypes are presented in Table 1.

The reconstructions are computed in parallel by several cores and/or computers in a cluster. The number of processors can be adapted to the desired throughput. The latency is more difficult to reduce below some limit, but it only affects the design of (distance to) the actuator that will take the action according to the quality control decision.

Adapting the system to larger objects is easy, since scaling the structural components is all that is needed. Adaptation to smaller objects (reaching a higher resolution) can be achieved by reducing the system or using a different focal length for the camera optics.

## 5. 3D Metrics

As discussed above (see Figure 4), the system described can obtain an approximated reconstruction of the objects captured. In the best case, it can attain the object’s visual hull. Therefore, the approximation at each point is bounded and can be computed.

A precise estimation of the best-case and the expected reconstruction errors can be useful or even mandatory for some applications. For example, to check object measurements in a quality control system, it will be necessary to take into account the measurement locations and whether the visual hull at those points is sufficiently close to the real object.

Since the proposed system is still under development, a formal characterization of its measurement capabilities and limits according to international standards has not yet been attempted. Metrology norms for coordinate measuring machines VDI/VDE (Association of German Engineers) 2617 and 2634, and ISO 10360 are not directly applicable to our system but offer characteristic tests that can probably be adapted. In particular, VDI/VDE 2634 Part 3 applies to scenarios with multiple views or multiple depth images from different relative positions of the sensors and the measured object and includes the consideration of plane-spacing errors, sphere-spacing errors, probing errors (form and size), length measurement errors, limit values (maximum permissible errors), diagonal of the specified measuring volume, etc.

Other norms are the American ASME (American Society of Mechanical Engineers) B89 for non-contact scanning probes, the European DIN (German Institute for Standardization) 32877:2000-08 for optoelectronic measurement of form, profile and distances and, in the domain of GD&T( Geometry, Dimensioning and Tolerances), there are other ANSI/ASME and ISO standards (Y14.5.1, Y14.8, Y14.41, Y14.43, Y14.45, ISO 286, ISO 1101, ISO 1119, ISO 1660, ISO 3040, ISO 5458, ISO 5459, ISO 14405, etc.).

Assessing the accuracy of the system according to the tests and measurements of these norms, when applicable, is one of our future targets, but, in the current stage of development of the system, we have pursued a first characterization of some basic global and local metrics, computing error bounds and intensity maps. Global metrics show differences among the meshes (volume, surface, etc.); local metrics reveal specific differences between the points of the compared object and intensity maps allow an easy 3D visualization of those differences.

### 5.1. Global Metrics

Global metrics are associated with features as surface or volume, summarizing information about the whole object.

- Mesh surface: Let *M* be a triangle mesh and *t* a triangle in *M*. The total mesh surface is computed as the sum of the areas of all the triangles of the mesh:
  (4) $$S_{M}=\sum_{t\in M}\mathrm{Area}\left(t\right).$$
- Mesh volume: The triangle mesh volume is calculated as the signed volume of all the tetrahedra made up of every triangle and the origin. Let $H_{t}$ be the tetrahedron defined by a given triangle t={a,b,} and the origin *O* (we assume that the object is normalized so that its center of masses is at the origin). Thus, the volume $v_{T}$ of a single tetrahedron is computed as:
  (5) $$v_{T}\left(H_{t}\right)=\frac{det\left[\begin{array}{c} a-b\\ b-c\\ c-O \end{array}\right]}{6}.$$
  *M* being a closed triangle mesh, the mesh volume $v_{M}$ will be calculated as the sum of the signed tetrahedron volume,
  (6) $$v_{M}=\sum_{t\in M}v_{T}\left(H_{t}\right).$$
- Octree volume: Let *x* be a voxel and $X_{I}$ the set of voxels that are inside the object. The octree volume is then calculated as the sum of the cube volumes belonging to $X_{I}$
  (7) $$V_{O}=\sum_{x\in X_{I}}\mathrm{Cube}\_\mathrm{volume}\left(x\right).$$

### 5.2. Local Metrics

Given two aligned meshes, local metrics compute differences based on pairs of points, each from a mesh [34]:

- Hausdorff distances: Let e(p,M) represent the distance from a mesh point p to a 3D object *M*:
  (8) $$e\left(p,M\right)=\min_{p^{\prime }\in M}\delta \left(p,p^{\prime }\right),$$
  δ being the Euclidean distance. Then, the asymmetric Hausdorff distance between two 3D objects *M* and $M^{\prime }$ is
  (9) $$H_{a}\left(M,M^{\prime }\right)=\max_{p\in M}e\left(p,M^{\prime }\right),$$
  and the symmetric Hausdorff distance also known as *maximum geometric error* is defined as follows:
  (10) $$H_{s}\left(M,M^{\prime }\right)=\max\left\{H_{a}\left(M,M^{\prime }\right),H_{a}\left(M^{\prime },M\right)\right\}.$$
- Mean distance:—also known as *mean geometric error* and is, in fact, another Hausdorff distance. It is defined as:
  (11) $$H_{\mathrm{mean}}\left(M,M^{\prime }\right)=\frac{1}{\mathrm{Area}(M)}\int_{M}e\left(p,M^{\prime }\right)dM.$$

### 5.3. Intensity Images

To represent the numerical differences in points pairs, representing the errors (distances) between the points of the real and the reconstructed objects, we use grayscale: white areas represent minimum errors and black areas maximum errors. These intensity images give visual information of the maximum reconstruction inconsistency, allowing the user to study the limits of the performance of the system.

Let p be a point belonging to an object *M* considered as the reference object, and let $M^{\prime }$ be a test object. Thus, the intensity value of p is calculated as:

(12) $$I\left(p,M,M^{\prime }\right)=\frac{e(p,M^{\prime })}{H_{s}\left(M,M^{\prime }\right)}.$$

## 6. Experiments and Results

### 6.1. Calibration

A set of spherical objects was used to test the proposed global calibration scheme (see Figure 13). After every capture, the images were segmented and the projections of the sphere centers on the camera sensor planes were estimated as the centroid of the pixels marked as an object in each image. Other alternatives involving approximating a circle to the image, according to several circle fitting algorithms, were tried too, but the pixel centroid method showed the most accurate results.

**Figure 17:**
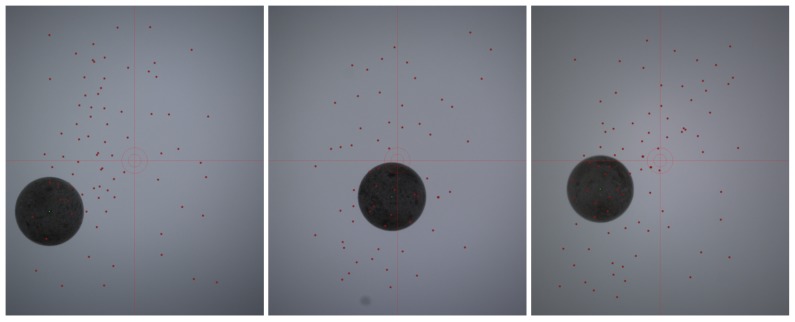
Distribution of the centers found for the first three cameras in the captures corresponding to 79 different launches of the calibration sphere.

A test was conducted with 79 captures of the calibration object. To avoid the object from following the same path in each capture and thus maximizing the variability, the sequencer hatch was released before the object was stabilized inside it. Thus, a set of acquisitions with the spheres widely distributed in the capture volume was obtained (see Figure 17).

The calibration error was evaluated at each iteration of the algorithm by means of two metrics: on the one hand, we computed the *reciprocal error* [27] for each camera. This metric averages the distances between the sphere centers and the projections of the computed 3D points ($p_{k}^{\prime }$) on the image space, giving a measure of the calibration accuracy for each camera. The *mean reciprocal error* is calculated as the average of the reciprocal errors of all cameras.

On the other hand, a global mean distance *m* was computed as the average of the distances from each point $p_{k}^{\prime }$ to the corresponding camera projection rays $r_{ki}^{\prime }$. It was computed as:

(13) $$m=\frac{1}{s\cdot c}\sum_{k=1}^{s}\sum_{i=1}^{c}\delta \left(p_{k}^{\prime },{\tilde{r}}_{ki}\right),$$

where ${\tilde{r}}_{ki}=c_{ki}+\left(\left(c_{ki}-p_{k}^{\prime }\right)\cdot d_{ki}\right)d_{ki}$ is the nearest point in $r_{ki}^{\prime }$ to $p_{k}^{\prime }$, *c* represents the number of cameras (16 in the system studied) and *s* is the number of capture sets (79 in this experiment). The more consistent the world system coordinates of the cameras are, the lower the value of *m* will be.

**Figure 18:**
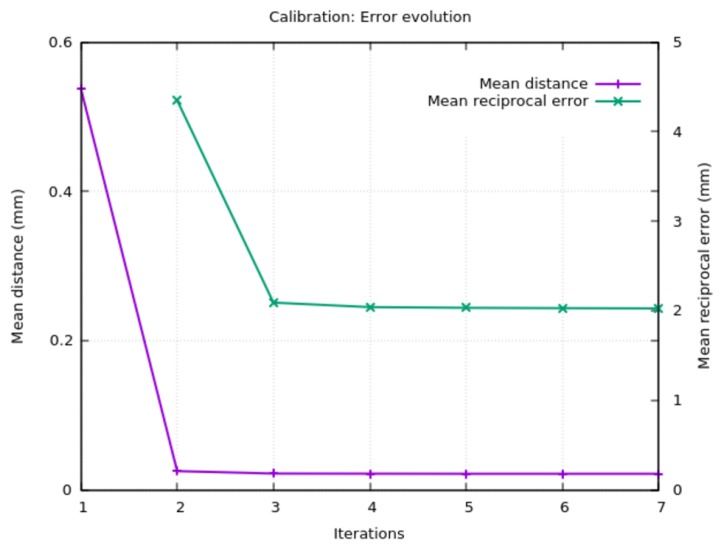
Mean distance and mean reciprocal error evolution during calibration. The relative progression of the mean reciprocal error was used as the stopping criterion.

As it can be seen in Figure 18, the mean distance and the reciprocal error converge in a few iterations. As stated in Section 3.1, an initial estimation of the camera position is needed. If this estimation is good, the algorithm converges faster.

It is interesting to point out that allowing the algorithm to evolve more iterations results in a tendency to “move the cameras” closer to the center of the capture volume. The correct orientation is maintained, but this will have an impact on the scale of the reconstructed objects that has to be corrected. To minimize this effect, several convergence functions based on the mean distance and the reciprocal error were tested as the stopping criteria. The estimation of the relative progression θ of these measures was computed as:

(14) $$\theta =|\frac{e-e^{\prime }}{e}|,$$

where *e* is the current error value (mean distance or mean reciprocal error), and $e^{\prime }$ is the value of the last iteration. A threshold of 0.01 for θ using the mean reciprocal error was employed as the stopping criterion.

Another set of experiments intended to estimate the minimum number of captures needed for the algorithm to give accurate calibrations was performed. The results indicate that from seven captures, assuming that the captured spheres are randomly distributed, the accuracy is stable.

**Figure 19:**
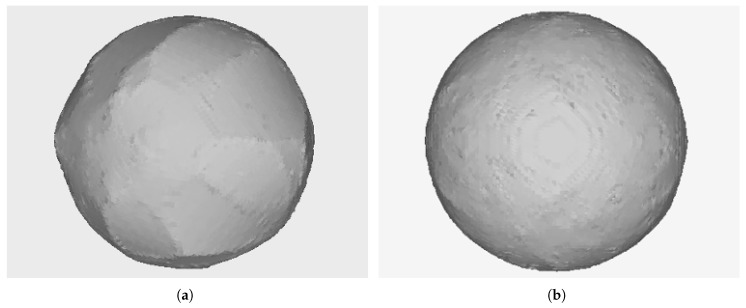
A 6-millimeter spherical object reconstructed without and with the proposed calibration scheme. (**a**) Local camera calibration. (**b**) Proposed global calibration algorithm.

**Figure 20:**
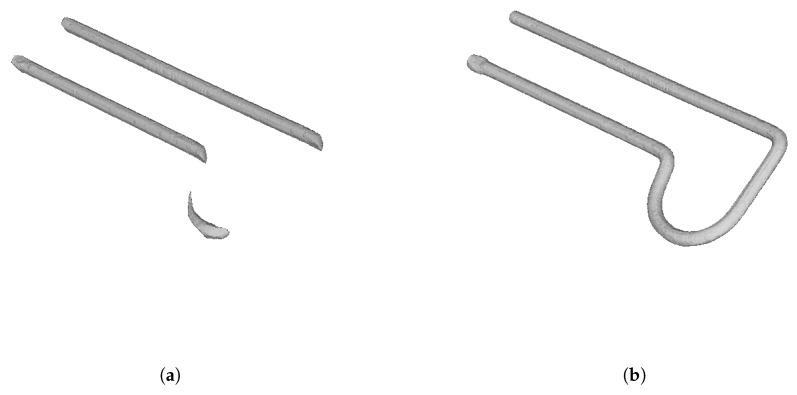
A thin object reconstruction employing: (**a**) local camera calibration and (**b**) the proposed global calibration algorithm.

Finally, Figure 19 and Figure 20 show real objects reconstructed by the system calibrated with a local camera calibration method and with the proposed global algorithm.

### 6.2. Metrics

The last analysis and sets of experiments are intended to measure and characterize the differences between the real objects and their reconstructions. The tests involved a set of virtual objects reconstructed by the algorithms used in the real system. A number of synthetic captures of these objects were obtained by means of the software *povray* [35]. In this analysis, the goal was to isolate possible errors induced by the physical acquisition system and the segmentation process and characterize the theoretical limitations of the system.

**Figure 21:**
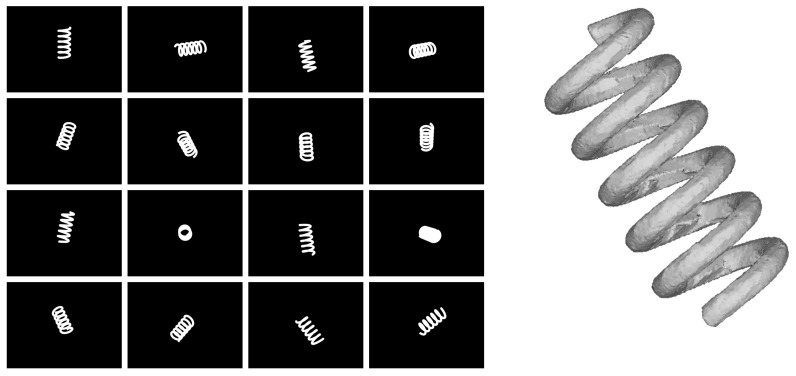
(**Left**) synthetic silhouettes from the 16 views of a virtual spring; (**Right**) reconstructed spring.

In the real system, the free-falling objects are captured at random orientations and the alignment with the cameras is always different. Accordingly, the synthetic captures of the virtual objects follow the same behavior and the images for each virtual camera are provided to the reconstruction software, which generates a 3D surface using the method described in Section 2. In Figure 21, an example of synthetic captures and reconstruction of a virtual spring can be shown.

#### 6.2.1. Computing the Theoretical Surfaces

The theoretical 3D points from the synthetic shapes were obtained and the tool *meshlab* [36] was used to generate the meshes from the point sets. Table 2 shows the number of points and triangles of the virtual objects used in the experiments. The resolution used is 200 points per revolution or per face side.

To obtain a good error estimation, the variable orientation of the free-falling objects was simulated rotating the virtual objects on the *x*- and *y*-axes. Because the objects are captured in the center of the system and the cameras are distributed around it, rotating only in those axes will be enough to cover all the possibilities. A 6-degree step on the *x*- and *y*-axes in a [0,180] range was chosen, thus a total of 900 different views of every object are taken into account.

#### 6.2.2. Global Metrics

The surface error is the difference of the theoretical mesh surface defined in Equation (4) and the reconstructed mesh surface. This error can be shown in Table 3. The error values were obtained by averaging the surface differences of the rotation of every object. The *cube*, the *sphere*, and the *cylinder* gave the best results, while the *spring* gave the worst value. This is a consequence of the hidden volume in the inner side of the spring produced by the carving process. The problem can be minimized by increasing the number of cameras (see Figure A1).

The volume errors computed from the octree volume defined in Equation (7) and the mesh volume defined in Equation (5) can be observed in Table 4. The octree volume errors are bigger than the mesh volume errors because the octree represents a discretized version of the reconstructed objects (see Figure 8); this agrees with the results of Szeliski in [15]. Once again, the *sphere* showed the best results and the *spring*, the worst.

#### 6.2.3. Local Metrics

The reconstructed objects and their models should be first aligned to compute the local metrics. Thus, an *Iterative Closest Point* algorithm [37] was employed for this purpose. Table 5 resumes the results obtained. All of the objects show a small average error ($H_{\mathrm{mean}}$) defined in Equation (11), though the maximal error values ($H_{s}$) defined in Equation (10) are larger for the *cube* and the *cylinder*. In this case, both objects present flat sides. This behavior was expected considering the nature of the carving process applied.

#### 6.2.4. Intensity Images

**Figure 22:**
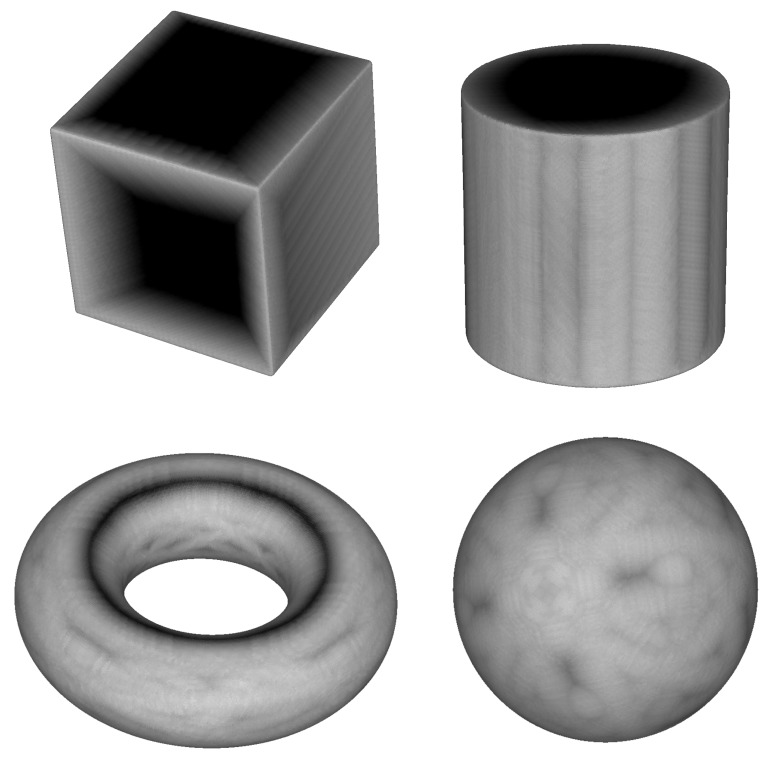
Intensity maps showing the differences between synthetic objects and their reconstructed counterparts.

Finally, intensity images showing the differences between the models and their reconstructions were computed (see Figure 22). The models were portrayed in grayscale, with white meaning no error and black meaning the largest error. The results agree with the previous conclusions: regions close to the sharp edges are reconstructed better than flat regions. This is due to the fact that they are closer to the image silhouettes.

Using these images, meaningful information can be obtained to determine the suitable candidate regions of each model for each specific task.

## 7. Conclusions

In the present work, a complete system able to capture objects and reconstruct them in 3D based on simultaneous captures from different cameras has been presented. Since the system is designed so that the object under inspection is in free fall, without a supporting surface, there are no hidden parts of it and the reconstruction of its visual hull is complete. The visible surface of the whole object is also captured. In the presented prototypes, 16 cameras with 5 Mpixels each have been used for the image acquisition, though the system can easily be adapted to work with a different number of image sensors.

A specific calibration algorithm has been developed to improve the accuracy in relation to conventional calibration algorithms, since the camera configuration does not allow that all cameras see a set of common features in a conventional calibration target.

The reconstruction accuracy of the prototypes has been analyzed with several examples and experiments. The computational cost is high, but reasonable for the system to be employed in 3D inspection in different industrial machine vision tasks.

Experiments have also been performed to evaluate the quality that can be expected for the reconstruction process in new parts, by means of global and local metrics and intensity maps. Those measures show that errors are mainly located in object flat areas, where the carving process does not provide information. To minimize this problem, more cameras can be installed, or several captures of the same object can be made. Finally, intensity maps are shown as a useful tool to select the regions of a specific object that offer a better quality of reconstruction.

## 8. Future Works

The system presented has a very wide spectrum of applications and is useful in very different industry sectors. We are investigating the quality requirements that are most valued in each manufacturing field by collaborating with end users in a number of different industries. Some elements of the design will probably be improved and refined once the system is tested in operational settings. The immediate areas of future work are:

- **Calibration:** The current calibration method gives rise to very accurate position parameters for the cameras and optics, but for objects with very clear, shiny or dark surfaces, it could be affected by segmentation variability (a few pixels could toggle from object to no-object or vice versa, depending on the contrast object/background, the position in the acquisition volume and the precision of the focus). We are investigating how, in the same calibration process, the absolute target size could be integrated in the optimization to be able to reach a high accuracy also in the scale estimation of the reconstructed objects.
- **Surface Quality Control:** The proposed system allows the 3D reconstruction of the object volume, but also captures its whole surface. Integrating the volume with the surface information will allow the system to check the quality of the surface at the same time as the correctness of the 3D geometry.
- **Several Captures:** The number of cameras in the system is a trade-off between accuracy and cost. Keeeping constant that number and acquiring the same object several times in a row will (by a mechanism that picks it up after each cycle and elevates it to be thrown again into the system) allow for iterative refinements of the reconstruction effectively obtaining a result equivalent to having a larger number of cameras. The first implementations of that process are currently being evaluated.

## Figures and Tables

**Table 1:** Current prototypes specifications.

	Prototype Size	
	ϕ 500 mm	ϕ 1000 mm
#Cameras	16	
Sensors	Sony IMX264 5.1 MP	
Sensor size	2448 × 2048	
Max object size	30 mm	30 mm
Resolution	20 um/pix	70 um/pix
Throughput	Up to 23 objects/s	
Latency	1–10 s	

**Table 2:** Theoretical surfaces.

Shape	Vertices	Triangles
Sphere	19,802	39,600
Cube	240,002	480,000
Cylinder	40,202	80,400
Spring	200,202	400,400
Torus	80,402	160,400

**Table 3:** Surface difference measurements.

Shape	Err (%)
Sphere	2.6
Cube	2.4
Cylinder	2.5
Spring	22.6
Torus	4.7

**Table 4:** Volume difference measurements.

Shape	Octree Err (%)	Mesh Err (%)
Sphere	8.1	1.2
Cube	17.6	10.7
Cylinder	9.9	3.6
Spring	35.9	16.2
Torus	10.1	2.5

**Table 5:** Local error measurements in millimeters. Objects have a size of 50 mm in their largest dimension.

Shape	$H_{s}$	$H_{\mathrm{mean}}$
Sphere	1.56	0.06
Cube	6.23	0.14
Cylinder	6.35	0.16
Spring	2.31	0.07
Torus	2.12	0.06
